# New prognostic scoring system for mortality in idiopathic pulmonary fibrosis by modifying the gender, age, and physiology model with desaturation during the six-minute walk test

**DOI:** 10.3389/fmed.2023.1052129

**Published:** 2023-01-25

**Authors:** Jae Ha Lee, Ji Hoon Jang, Hang-Jea Jang, Song Yee Kim, Man Pyo Chung, Hongseok Yoo, Sung Hwan Jeong, Jin Woo Song, Hong Lyeol Lee, Sun Mi Choi, Young Whan Kim, Yong Hyun Kim, Sung Woo Park, Jong Sun Park, Yangin Jegal, Jongmin Lee, Soo-Taek Uh, Tae-Hyung Kim, Yee Hyung Kim, Beomsu Shin, Hyun-kyung Lee, Sei-Hoon Yang, Hyun Lee, Sang-Heon Kim, Eun-Joo Lee, Hye Sook Choi, Hyung Koo Kang, Eun Young Heo, Won-Yeon Lee, Moo Suk Park

**Affiliations:** ^1^Division of Pulmonology and Critical Care Medicine, Department of Internal Medicine, Inje University Haeundae Paik Hospital, Inje University College of Medicine, Busan, Republic of Korea; ^2^Division of Pulmonology, Department of Internal Medicine, Severance Hospital, Yonsei University College of Medicine, Seoul, Republic of Korea; ^3^Division of Pulmonary and Critical Care Medicine, Department of Medicine, Samsung Medical Center, Sungkyunkwan University School of Medicine, Seoul, Republic of Korea; ^4^Department of Allergy, Pulmonology and Critical Care Medicine, Gil Medical Center, Gachon University, Incheon, Republic of Korea; ^5^Division of Pulmonary and Critical Care Medicine, Asan Medical Center, University of Ulsan College of Medicine, Seoul, Republic of Korea; ^6^Department of Internal Medicine, School of Medicine, Inha University, Incheon, Republic of Korea; ^7^Division of Pulmonary and Critical Care Medicine, Department of Internal Medicine, Seoul National University College of Medicine, Seoul, Republic of Korea; ^8^Division of Allergy and Pulmonology, Department of Internal Medicine, Bucheon St. Mary’s Hospital, The Catholic University of Korea School of Medicine, Bucheon-si, Republic of Korea; ^9^Division of Allergy and Respiratory Medicine, Department of Internal Medicine, Soonchunhyang University Bucheon Hospital, Bucheon-si, Republic of Korea; ^10^Division of Pulmonary and Critical Care Medicine, Department of Internal Medicine, Seoul National University Bundang Hospital, Seongnam-si, Republic of Korea; ^11^Division of Pulmonary Medicine, Department of Internal Medicine, Ulsan University Hospital, University of Ulsan College of Medicine, Ulsan, Republic of Korea; ^12^Division of Pulmonary, Allergy and Critical Care Medicine, Department of Internal Medicine, College of Medicine, Seoul St Mary’s Hospital, The Catholic University of Korea, Seoul, Republic of Korea; ^13^Division of Pulmonary and Allergy Medicine, Department of Internal Medicine, Soonchunhyang University Hospital, Seoul, Republic of Korea; ^14^Division of Pulmonary and Critical Care Medicine, Hanyang University Guri Hospital, Hanyang University College of Medicine, Guri, Republic of Korea; ^15^Division of Pulmonary and Critical Care Medicine, Department of Internal Medicine, Kyung Hee University Hospital at Gangdong, Kyung Hee University School of Medicine, Seoul, Republic of Korea; ^16^Department of Internal Medicine, Samsung Changwon Hospital, Sungkyunkwan University School of Medicine, Changwon, Republic of Korea; ^17^Division of Pulmonary and Critical Care Medicine, Department of Internal Medicine, Inje University Busan Paik Hospital, Busan, Republic of Korea; ^18^Division of Pulmonary, Department of Internal Medicine, College of Medicine, Wonkwang University, Iksan, Republic of Korea; ^19^Department of Internal Medicine, Hanyang University College of Medicine, Seoul, Republic of Korea; ^20^Division of Respiratory and Critical Care Medicine, Department of Internal Medicine, Korea University Anam Hospital, Korea University College of Medicine, Seoul, Republic of Korea; ^21^Department of Pulmonary and Critical Care Medicine, Kyung Hee University Medical Center, School of Medicine, Kyung Hee University, Seoul, Republic of Korea; ^22^Division of Pulmonology and Critical Care Medicine, Department of Internal Medicine, Inje University Ilsan Paik Hospital, Inje University College of Medicine, Busan, Republic of Korea; ^23^Division of Pulmonary and Critical Care Medicine, Department of Internal Medicine, Seoul Metropolitan Government-Seoul National University Boramae Medical Center, Seoul, Republic of Korea; ^24^Department of Internal Medicine, Yonsei University Wonju College of Medicine, Wonju, Republic of Korea

**Keywords:** idiopathic pulmonary fibrosis, interstitial lung disease, mortality, prognosis, six-minute walk test

## Abstract

**Background:**

Idiopathic pulmonary fibrosis (IPF) is a progressive fibrosing interstitial lung disease (ILD) with variable and heterogeneous clinical course. The GAP (gender, age, and physiology) model had been used to predict mortality in patients with IPF, but does not contain exercise capacity. Therefore, our aim in this study was to develop new prognostic scoring system in the Korea IPF Cohort (KICO) registry.

**Materials and methods:**

This is a retrospective study of Korean patients with IPF in KICO registry from June 2016 to August 2021. We developed new scoring system (the GAP6) based on the GAP model adding nadir saturation of percutaneous oxygen (SpO_2_) during six-minute walk test (6MWT) in the KICO registry and compared the efficacy of the GAP and the GAP6 model.

**Results:**

Among 2,412 patients in KICO registry, 966 patients were enrolled. The GAP6 model showed significant prognostic value for mortality between each stage [HR Stage II vs. Stage I = 2.89 (95% CI = 2.38–3.51), HR Stage III vs. Stage II = 2.68 (95% CI = 1.60–4.51)]. In comparison the model performance with area under curve (AUC) using receiver operating characteristic (ROC) curve analysis, the GAP6 model showed a significant improvement for predicting mortality than the GAP model (AUC the GAP vs. the GAP6, 0.646 vs. 0.671, *p* < 0.0019). Also, the C-index values slightly improved from 0.674 to 0.691 for mortality.

**Conclusion:**

The GAP6 model adding nadir SpO_2_ during 6WMT for an indicator of functional capacity improves prediction ability with C-index and AUC. Additional multinational study is needed to confirm these finding and validate the applicability and accuracy of this risk assessment system.

## Introduction

Idiopathic pulmonary fibrosis (IPF) is a typical and progressive chronic fibrosing interstitial lung disease (ILD) with a highly variable clinical course and poor outcomes (1). Despite recent advances, including anti-fibrotic agents, and increasing awareness of IPF, its mortality rate is still high, and the median survival time is only 2.5–4 years (2–4). Moreover, the clinical course and prognosis vary widely according to the presence of acute exacerbation, comorbidities, disease severity, and availability and side effects of anti-fibrotic agents (5–7).

Staging systems of disease severity are crucial and useful for determining prognosis and guiding management decisions. Several clinical prediction models have been developed for patients with IPF, and the gender, age, and physiology (GAP) model is most commonly used (8–10). The GAP model is simple and convenient to use and has been demonstrated to be reliable for predicting survival in previous studies (9, 11). However, the GAP model has some limitations. In a validation study of the GAP model, there was a lack of discriminative performance for predicting prognosis according to stage or over a long term (12, 13). Also, the GAP model is based on gender, age, and lung function data as baseline predictors without considering other important predictors, including exercise capacity and hypoxemia. The six-minute walk test (6MWT) is a basic test recommended in international guidelines due to its simplicity, and desaturation during 6 MWT is a strong predictor of mortality in IPF patients (5, 14). Therefore, our aim in this study was to develop a new prognostic scoring system modifying the GAP model with desaturation during the 6MWT using data from the Korea IPF Cohort (KICO) registry.

## Materials and methods

### Study subjects

Patients with IPF included in the KICO registry from June 2016 to August 2021 were enrolled in this retrospective study. A total of 23 universities and teaching hospitals in Korea was involved in the KICO registry, and IPF diagnosis was based on multidisciplinary discussion (MDD) among health care professionals, including a pulmonologist, radiologist, and pathologist, according to the criteria of the American Thoracic Society (ATS)/European Respiratory Society (ERS) guideline (14, 15). Medical records of patients were reviewed using KICO web-based registry data.^1^ This study was approved by the Institutional Review Board of Haeundae-Paik Hospital (approval no. 2021-07-017), and the requirement for written informed consent was waived due to the retrospective nature of this study.

### Validation of the GAP model

Total GAP score was calculated by four clinical variables of gender (women: 0 point, man: 1 point), age (0–2 point), FVC (0–2 point), and DLco (0–3 point). The GAP stage was classified into three stages based on the total GAP score. Validation of the GAP model in the KICO registry was performed to evaluate the effectiveness of the new scoring system modifying the GAP model with desaturation during 6MWT. Total GAP score calculations and disease stage classifications were completed based on the criteria originally suggested by Ley et al. (9). We evaluated the 1-, 2-, and 3-year mortality rates at each stage or score based on the GAP index.

### Development of a new scoring system modifying the GAP model

Baseline values at IPF diagnosis were considered as predictors in this study. After Cox proportional hazards regression analysis to predict survival, desaturation during 6MWT was confirmed to be a statistically significant predictor in addition to variables of the GAP model. Thus, we selected and added desaturation during 6MWT to the GAP model and developed the new scoring system, known as GAP6, to predict mortality in IPF patients. We added the points for desaturation in the GAP6 model according to the coefficients of the Cox regression models, and a nomogram consisting of five variables with point contributions was created using the GAP6 model. Finally, we compared the efficacy for predicting prognosis between the GAP model and the GAP6 model using the C-index and area under the receiver operating characteristic (ROC) curve (AUC).

### Statistical analysis

Data are presented as frequency with percentage for categorical variables and mean ± standard deviation (SD) for continuous variables.

Overall survival probability was estimated with the Kaplan–Meier method. The difference between the three disease stages was assessed using the log-rank test. The time interval was measured from the day of diagnosis until death or last follow-up. Death from all causes was included. Univariate and multivariate Cox proportional hazards models were fit to examine the relationships between survival time and patient characteristics.

Nomogram development began by identifying patient characteristics predictive for overall survival in the multivariate Cox model. These characteristics were gender, age, forced vital capacity (FVC), diffusing capacity of the lung for carbon monoxide (DLco), and nadir saturation of percutaneous oxygen (SpO2).

Univariate and multivariate analyses using binary logistic regression were performed to identify prognostic factors independently related to 3-year mortality. In addition, to compare model performance, ROC curve analysis was performed to assess the sensitivity and specificity of the modified GAP score and staging system to predict 3-year mortality.

All statistical analyses were carried out using SPSS version 26.0 (IBM Corp. Released 2019. IBM SPSS Statistics for Windows, Version 26.0. Armonk, NY: IBM Corp.), R version 4.1.2 (R Core Team [2021]. R: A language and environment for statistical computing. R Foundation for Statistical Computing, Vienna, Austria. URL, https://www.R-project.org/), and MedCalc Statistical Software version 19.2.6 (MedCalc Software Ltd., Ostend, Belgium; https://www.medcalc.org; 2020). *p* < 0.05 was considered statistically significant.

## Results

### Study population and baseline characteristics

**Figure 1:**
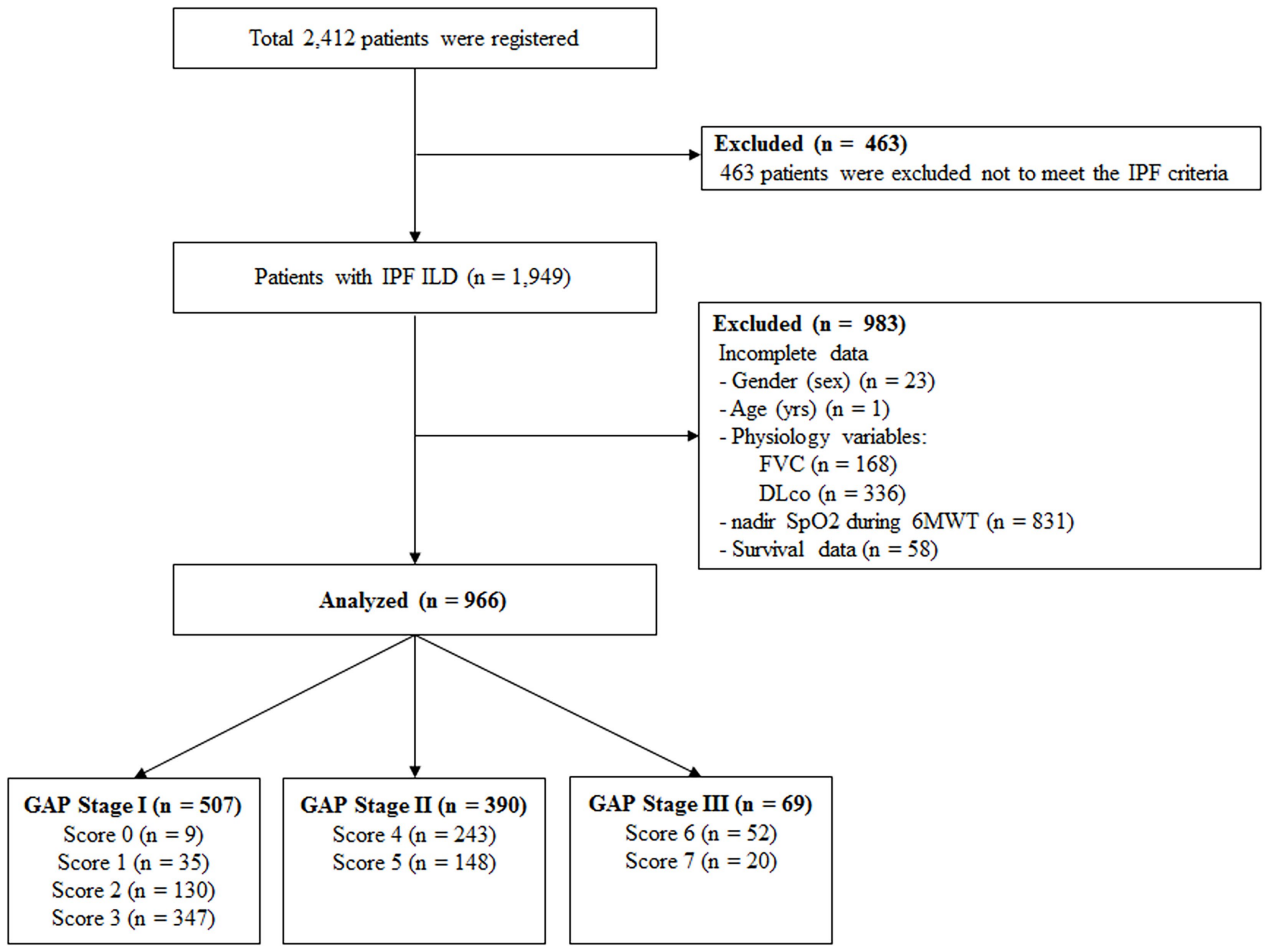
Overview of study design IPF, idiopathic pulmonary fibrosis; ILD, interstitial lung disease; FVC, functional vital capacity; DLco, diffusing capacity of the lung for carbon monoxide; SpO2, saturation of percutaneous oxygen; 6MWT, six-minute walk test; GAP, gender, age, and physiology.

A total of 2,412 patients was registered from June 2016 to August 2021 in the KICO registry (Figure 1). Of them, 463 patients were excluded because they did not meet the international criteria for IPF diagnosis after central and individual institutional MDD reviews. Additionally, among 1,949 patients with IPF, 222 with incomplete data for gender, age, physiologic variables, or survival data were also excluded. Finally, 966 patients were included in the analysis. The baseline characteristics of these patients are summarized in Table 1. The mean age was 71.2 years, and men (81.1%) were more common than women. The mean age at diagnosis of IPF was 68.2 years (men vs. women, 68.1 vs. 68.7 years; *p* = 0.301). Most patients exhibited a mild restrictive ventilator defect (FVC, % predicted between men and women, 73.9% vs. 74.9%, *p* = 0.471) and reduced DLco. During 6MWT, the mean distance was 412.7 m and the nadir SpO2 was 90.1%. More than two-thirds of the patients had been treated with anti-fibrotic agents, and there was no difference in treatment according to gender (men vs. women, 71.8% vs. 71.1%, *p* = 0.846).

**Table 1:** Baseline clinical characteristics of the patients.

Characteristics	All patients (*n* = 966)
Male	783 (81.1)
Age (years)	71.23 ± 7.69
Ever-smokers	714 (75.4)
Height (cm)	163.14 ± 8.36
Weight (kg)	64.71 ± 10.24
BMI (kg/m^2^)	24.40 ± 6.38
Radiologic pattern on HRCT	
UIP	470 (48.7)
Probable UIP	408 (42.2)
Home O2	159 (16.5)
mMRC	
Grade 0	195 (22.3)
Grade 1	333 (38.1)
Grade 2	255 (29.2)
Grade 3	73 (8.4)
Grade 4	17 (1.9)
Blood gas	
PaO_2_, mmHg	98.14 ± 38.64
BNP	260.46 ± 1230.83
Pulmonary function test	
FVC, % predicted	74.16 ± 15.47
DLco, % predicted	61.45 ± 18.99
FEV1/FVC, %	86.60 ± 15.78
Six-minute walk test	
Distance (m)	412.73 ± 184.97
Nadir SpO_2_, %	90.11 ± 6.78
RVSP, mmHg (*n* = 176)	31.15 ± 10.77
BAL fluid analysis	
Neutrophil, %	17.37 ± 19.83
Lymphocyte, %	13.60 ± 14.77
WBC	484.86 ± 773.51

Values are presented as mean ± standard deviation or number (%).

BMI, body mass index; HRCT, high resolution computed tomography; UIP, usual interstitial pneumonia; mMRC, modified medical research council; PaO_2_, partial pressure of oxygen; BNP, brain natriuretic peptide; %FVC, forced vital capacity % predicted; %DLco, diffusing capacity of the lung for carbon monoxide % predicted; FEV1/FVC, the ratio of forced expiratory volume at 1 s (FEV1) over forced vital capacity 9(FVC); SpO_2_, saturation of percutaneous oxygen; RVSP, right ventricular systolic pressure; BAL, bronchoalveolar lavage; WBC, white blood cell.

### Validation of the GAP model

**Figure 2:**
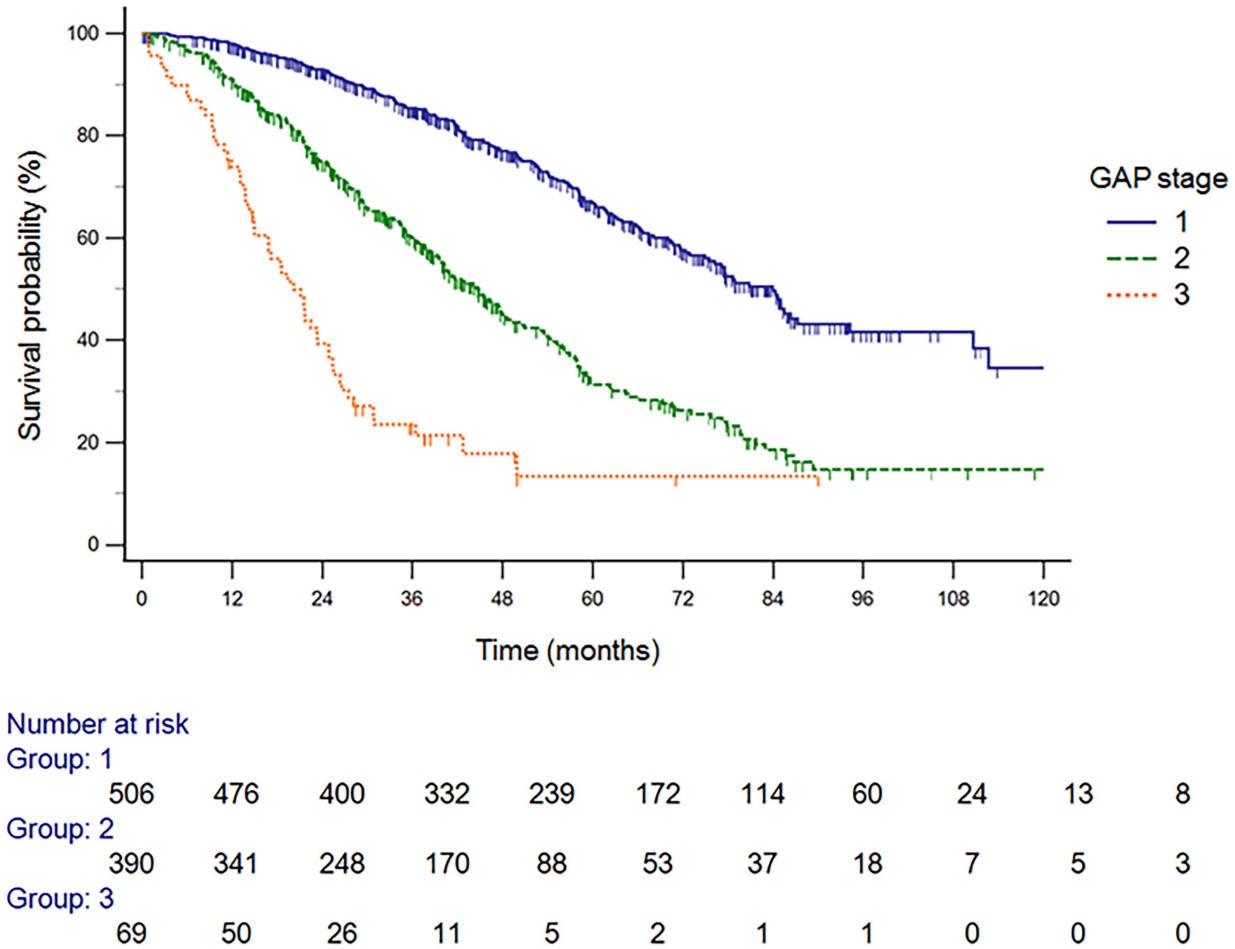
Survival probability analysis using Kaplan–Meier plotting, the 3-year mortality rates for the GAP stage I, II, and III groups GAP, gender, age, and physiology.

The GAP model revealed 507 patients with GAP stage I (52.5%), 390 patients with GAP stage II (40.4%), and 69 patients with GAP stage III (7.1%) (Table 2). The median duration of follow-up was 60.4 months. Of the 966 included patients, 440 (45.5%) died during the study period. The median time to death was 83.8 months (95% confidence interval [CI], 75.2–92.4 months). A total of 257 patients (26.6%) died within 3 years, and the observed cumulative mortality rate differed significantly according to GAP stage (log-rank test, *p* < 0.001). Survival was significantly different by disease stage (hazard ratio [HR] stage II vs. stage I, 2.52 [95% CI, 2.07–3.08]; HR stage III vs. stage II, 2.64 [95% CI, 1.52–4.61]) (Figure 2). In the analysis of survival probability using Kaplan–Meier plotting, the 3-year mortality rates for the GAP stage I, II, and III groups were 2.4, 6.5, and 50.1%, respectively. A statistically significant difference was found among the GAP stage I, II, and III groups (log-rank *p* < 0.001).

**Table 2:** Gender, age, and physiology (GAP) index and number (%) of patients.

Variable	GAP points	No. of patients
Gender		
Female	0	183 (18.9)
Male	1	783 (81.1)
Age (years)		
≤ 60	0	87 (9.0)
61–65	1	133 (13.8)
>65	2	746 (77.2)
Physiology		
FVC, % predicted		
> 75	0	455 (47.1)
50–75	1	456 (47.2)
< 50	2	55 (5.7)
DLco, % predicted		
> 55	0	590 (61.1)
36–55	1	292 (30.2)
≤ 35	2	84 (8.7)
Median (range)		
GAP stage		
Stage I	0–3	507 (52.5)
Stage II	4–5	390 (40.4)
Stage III	6–7	69 (7.1)

Values in parentheses are percentages.

GAP, gender, age, and physiology; FVC, forced vital capacity; predicted; DLco, diffusing capacity of the lung for carbon monoxide.

### Development of a new scoring system modifying the GAP model

The Cox proportional hazards regression analysis to verify the significance of nadir SpO2 (HR, 0.972; 95% CI, 0.960–0.984; *p* < 0.001) as a predictive variable showed that each prognostic factor except gender contributed to predict the survival in patients with IPF (Table 3). A higher nadir SpO2 significantly increased survival. Therefore, we added nadir SpO2 to the GAP6 model. According to the nomogram, the GAP6 model consisted of 5 variables. Information about the nomogram itself, such as the point–linear predictor unit mapping and total point–survival probability mapping, is shown in Supplementary Figure 1. We added the points for nadir SpO2 to the GAP6 model according to the coefficients of the Cox regression models, and the nomogram created using the GAP6 model consisted of five variables with point contributions (Table 4). The index score of the sum of the point contributions for each of the five characteristics was then calculated; an index score of 0–3 points indicated stage I (low risk), that of 4–6 points indicated stage II (intermediated risk), and that of 7–9 points indicated stage III (high risk) disease. In addition to variables in the GAP6 model, the presence of lung cancer (HR 1.88; 95% CI, 1.43–2.46, *p* < 0.001) and the use of antifibrotic agents (HR 0.76, 95% CI, 0.559–0.862, *p* = 0.001) during the follow-up period were significantly associated with mortality in the multivariable analysis. However, this analysis aims to develop predicting risk for mortality according to baseline characteristics in patients with IPF, the presence of lung cancer and the use of antifibrotic agents after diagnosis did not be included in the GAP6 model.

**Table 3:** Survival analysis with Cox proportional hazard model.

Variable	Univariate analysis			Multivariate analysis		
	HR	95% CI	*p*-Value	HR	95% CI	*p*-Value
Sex (M/F)	1.071	0.842–1.362	0.575	1.004	0.787–1.281	0.976
Age (years)	1.037	1.024–1.050	<0.001	1.040	1.027–1.053	<0.001
FVC, % predicted	0.962	0.955–0.968	<0.001	0.979	0.972–0.986	<0.001
DLco, % predicted	0.963	0.957–0.968	<0.001	0.975	0.969–0.982	<0.001
Nadir SpO_2_ (%)	0.951	0.943–0.959	<0.001	0.972	0.960–0.984	<0.001

M, male; F, female; FVC, forced vital capacity; DLco, diffusing capacity of the lung for carbon monoxide; SpO_2_, saturation of percutaneous oxygen.

**Table 4:** Prognostic index based on presence of factors.

Characteristic	Point contribution		
	0	1	2
Gender	Female	Male	–
Age (years)	≤60	61–65	>65
FVC, % predicted	>75	50–75	<50
DLco, % predicted	>55	36–55	≤35
Nadir SpO_2_ (%)	≥90	≤80–90	<80

The total points were distributed to the three stages as appropriate.

FVC forced vital capacity; DLco, diffusing capacity of the lung for carbon monoxide; SpO_2_, saturation of percutaneous oxygen.

**Figure 3:**
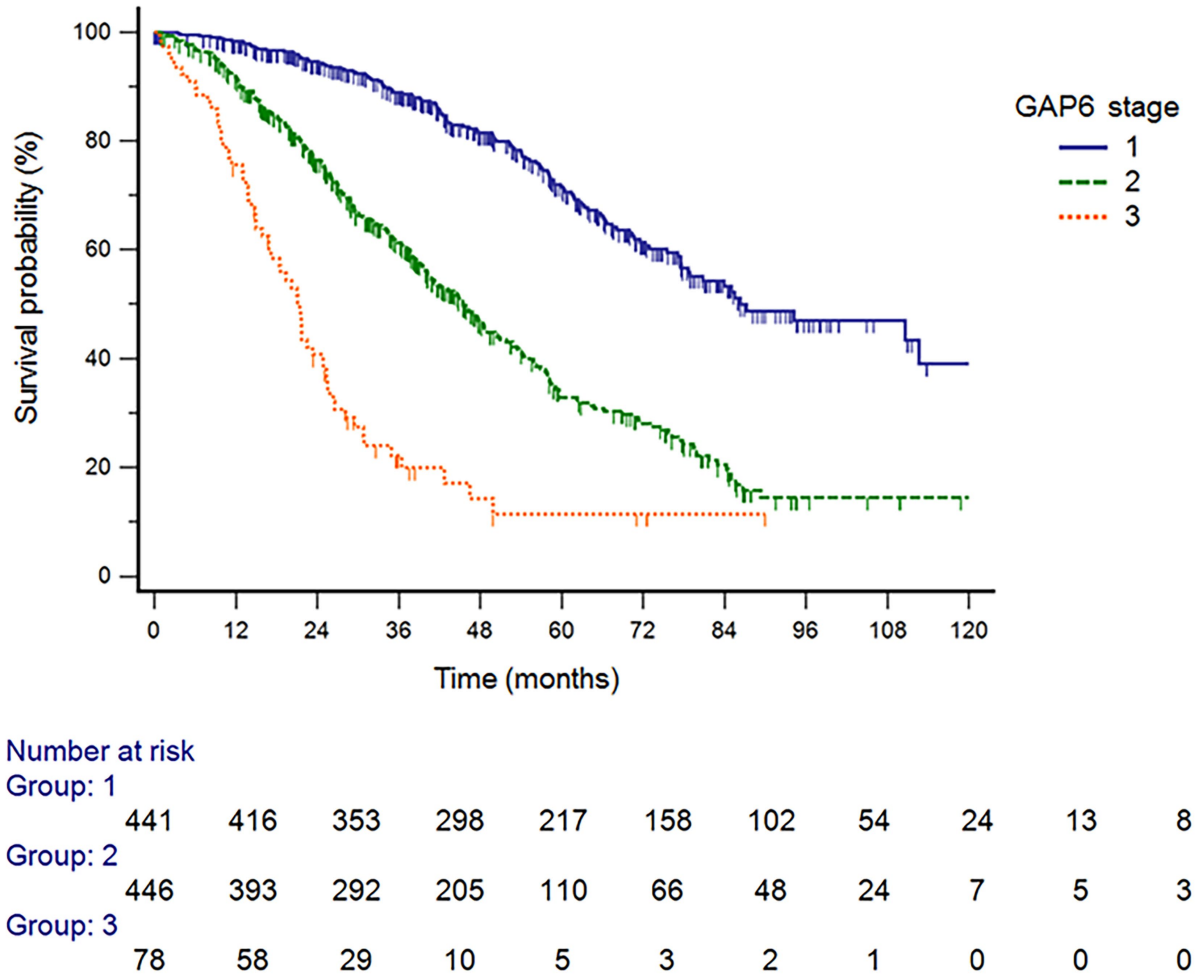
Survival probability analysis using Kaplan–Meier plotting, the 3-year mortality rates for the GAP6 stage I, II, and III groups GAP6, gender, age, and physiology with desaturation during six-minute walk test.

The GAP6 model revealed 442 patients with stage I (45.8%), 446 patients with stage II (46.2%), and 78 patients with stage III (8.1%) disease (Table 5). Figure 3 shows that survival differed significantly by disease stage (HR stage II vs. stage I, 2.89 [95% CI, 2.38–3.51]; HR stage III vs. stage I, 7.77 [95% CI, 4.66–12.96]; HR stage III vs. stage II, 2.68 [95% CI, 1.60–4.51]). In the analysis of survival probability using Kaplan–Meier plotting, the 3-year mortality rates for the modified GAP6 stages I–III groups were 2.3, 5.5, and 42.9%, respectively. A statistically significant difference was found among the modified GAP stages I–III groups (log-rank *p* < 0.001).

**Table 5:** GAP6 index and number (%) of patients.

Variable	GAP points	No. of patients
Gender		
Female	0	183 (18.9)
Male	1	783 (81.1)
Age (years)		
≤60	0	87 (9.0)
61–65	1	133 (13.8)
>65	2	746 (77.2)
Physiology		
FVC, % predicted		455 (47.1)
>75	0	456 (47.2)
50–75	1	55 (5.7)
<50	2	
DLco, % predicted		
>55	0	590 (61.1)
36–55	1	292 (30.2)
≤35	2	84 (8.7)
Nadir SpO_2_		
≥90	0	637 (65.9)
≤80–90	1	264 (27.3)
<80	2	65 (6.7)
GAP6 stage		
Stage I	0–3	442 (45.8)
Stage II	4–6	446 (46.2)
Stage III	7–9	78 (8.1)

Values in parentheses are percentages.

GAP6, gender, age, and physiology with desaturation during six-minute walk test; FVC, forced vital capacity; DLco, diffusing capacity of the lung for carbon monoxide; SpO_2_, saturation of percutaneous oxygen.

### Comparison of the GAP model and the GAP6 model using the KICO registry

The GAP model included 507 patients with stage I, 390 patients with stage II, and 69 patients with stage III disease. However, the difference between proportion of patients in each stage group between the GAP and GAP6 models were significant (McNemar–Bowker test *p* < 0.001). Among 507 patients with GAP stage I, 65 patients were allocated into the GAP6 stage II group, and 17 of 390 patients with GAP stage II were allocated into the GAP6 stage III group. Also, 8 of 69 patients with GAP stage III were allocated into the GAP6 stage II group. The 1-, 2-, and 3-year mortality rates (Kaplan–Meier estimates) at each stage based on the GAP and GAP6 models are shown in Table 6. Based on the GAP6 model, the C-index value was 0.691 (95% CI, 0.650–0.698), showing an improvement compared to the value calculated based on the GAP model (0.674 [95% CI, 0.667–0.715]). Therefore, with the use of the GAP6 model, the C-index value for mortality slightly improved from 0.674 to 0.691.

**Table 6:** Mortality rates for patients in different stages according to the original and modified GAP model.

Stage	Original GAP	GAP6
1-year mortality		
Stage I	0.8%	0.7%
Stage II	2.1%	1.6%
Stage III	17.4%	15.4%
2-year mortality		
Stage I	1.4%	1.6%
Stage II	4.1%	3.6%
Stage III	33.5%	29.6%
3-year mortality		
Stage I	2.4%	2.3%
Stage II	6.5%	5.5%
Stage III	50.1%	42.9%
C-index	0.674	0.691
(95% CI)	(0.650–0.698)	(0.667–0.715)

GAP, gender, age, and physiology; CI, confidence interval; GAP6, gender, age, and physiology with desaturation during six-minute walk test.

We compared the risk of death predicted by the GAP and GAP6 models with the observed mortality using calibration plots and goodness-of-fit statistics (Hosmer–Lemeshow test; e-Figure 2). Models for which expected and observed probabilities in GAP stages are similar are considered to be well calibrated. The solid line in Supplementary Figure 2 represents a perfect agreement between predicted and observed risks. We found that the GAP6 model predicted the 3-year mortality rate more accurately than the GAP model, although the predicted and observed risks were not significantly different across the three stages (Hosmer–Lemeshow *p* = 0.369 for GAP and *p* = 0.903 for GAP6).

**Figure 4:**
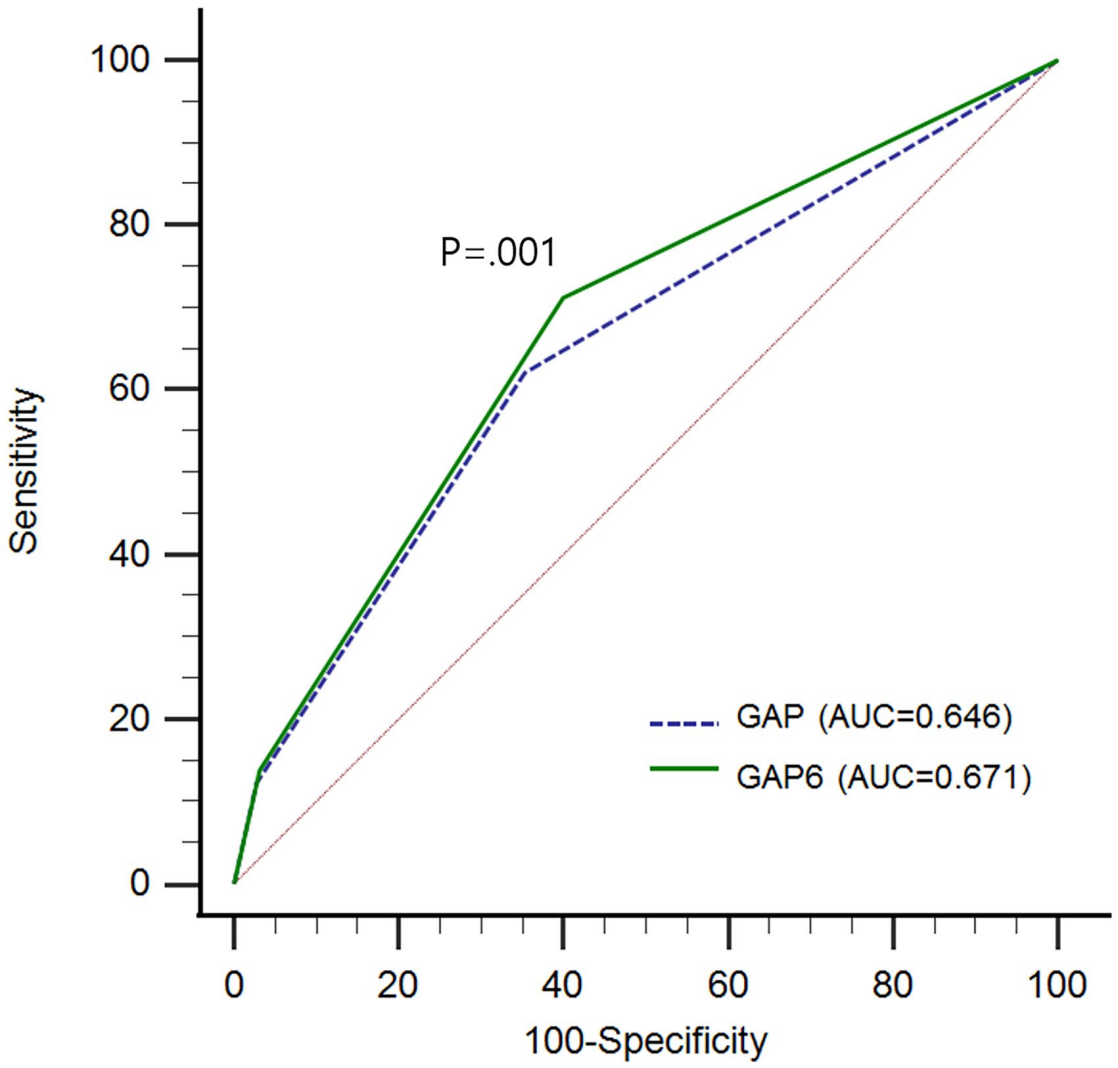
Comparison of predictive performance between GAP model and GAP6 with area under curves (AUCs) using receiver operating characteristic (ROC) curve analysis GAP, gender, age, and physiology; GAP6, gender, age, and physiology with desaturation during six-minute walk test; AUC, area under curve; ROC, receiver operating characteristic.

We compared the model performance with AUCs using ROC curve analysis. There was a significant difference between the GAP and the GAP6 models (AUC GAP vs. GAP6, 0.646 vs. 0.671; *p* < 0.0019; Figure 4).

## Discussion

The GAP model has been widely used to predict mortality in patients with IPF. However, the model has some limitations not to incorporate important variables for predicting mortality such as exercise capacity. Therefore, we developed a GAP6 model for the Korean population by adding nadir SpO2 as a predictor variable when calculating the GAP score. The GAP6 model improves the prediction ability with C-index and AUC.

The 6MWT is a practical and objective measure of functional exercise capacity, easy to perform, and reproducible (16, 17). In terms of IPF, exercised capacity, represented by distance and desaturation during 6MWT, is severely reduced due to the nature of IPF, including an abnormal gas-exchange response characterized by a significant decrease in arterial oxygen and increased differences between oxygen concentration in the alveoli and arterial system in the efficiency of alveolar ventilation (18, 19). In previous research, desaturation during 6MWT has been reported to be a significant predictor of mortality in patients with IPF (20, 21). Therefore, we hypothesized that nadir SpO2 representing desaturation is an important predictor of mortality and developed a new scoring system, GAP6, by adding nadir SpO2 to the GAP model.

In general, gender had been considered a significant predictor for mortality in patients with IPF (22, 23). However, in this study, gender did not trigger a statistically significant difference as a predictor for mortality in the Cox proportional hazards regression analysis and nomogram in the GAP model. Recent studies support this result of our investigation. Estrella et al. reported in 608 patients from the IPF national registry of the Spanish Respiratory Society that there was no statistically significant difference in mortality in men vs. women (HR, 1.5; 95% CI, 0.94–2.3, *p* = 0.092) (24). Lucile et al. in 246 patients from a French national multicenter prospective cohort demonstrated that women appear to be older with less frequent history of smoking and occupational exposures but survival comparable to that of men (HR, 0.85; 95% CI, 0.58–1.25, *p* = 041) (25). We suggested that no gender differences in age at diagnosis, lung function, or adherence to anti-fibrotic agents might be the reason for the same mortality rates of men and women in this study.

In the KICO registry, the 1- and 2-year mortality rates were significantly lower than those reported by Ley et al. in 2012 (2). A recent study of an IPF cohort with 562 patients showed a result similar to that of our study (26). The reason for such a result of lower mortality rate might be that, as anti-fibrotic agents were developed and widely employed in real-world practice, more patients were diagnosed actively at an early stage, resulting in the opportunity to receive appropriate treatment. Therefore, a new method for prognosis reflecting the real-world situation is warranted in the new era of anti-fibrotic agents.

In this study, the GAP6 model showed a better ability to predict mortality with C-index and AUC improvement than the GAP model. In the KICO registry, the proportion of anti-fibrotic agent use was higher than previously reported in other registry studies (10, 26, 27). Also, we added nadir SpO2 representing desaturation during the 6MWT to reflect functional capacity, which resulted in a statistically significant reclassification of patients in stages II and III. We assumed that 3-year mortality in the GAP model was overestimated due to a lack of consideration of functional capacity and the use of anti-fibrotics in the KICO registry; therefore, GAP6 is more useful to predict 3-year mortality. In the reality of increased and generalized anti-fibrotics use, we hope the result of our study will be more meaningful.

In addition to predictors of gender, age, physiology, and functional capacity in GAP6, there are several prognostic factors of mortality in patients with IPF (8, 28–31). In the Cox proportional hazards regression analysis in this study, body mass index (BMI), modified dyspnea scale of the Medical Research Council, and distance during 6MWT were independent prognostic factors of mortality. Also, the GAP6 model does not imply sequential change or decline of physiology and variables of 6MWT. Therefore, development of a new prognosis scoring model in IPF composed of other variables or sequential changes of existing variables is needed.

There are some limitations to this study. First, this was a retrospective study involving a single Korean cohort, and this might call into question the generalization of our findings to other cohorts. However, baseline and clinical characteristics, including a high rate of anti-fibrotic agent use, were similar to those of other recent cohort studies (24, 32, 33). The KICO registry includes recent data reflecting current real-world trends of diagnosis and anti-fibrotic agent use. Therefore, this study might be helpful in many ongoing and future cohort studies of patients with IPF. Second, we used nadir SpO2 as an indicator of desaturation during 6MWT. There was no consensus on the predictor of desaturation during 6MWT. Previous studies showed that desaturation defined as a ≥ 4% decrease in pre-exercise SpO2 during 6MWT is a significant predictor of mortality (20, 34). Since there were no data on pre-exercise SpO2 in the KICO registry, this study has the limitation that the relative decline of SpO2 between baseline and nadir could not be used as a predictor for desaturation. Third, we developed the GAP6 model based on the GAP model, adding desaturation to validate and compare the efficacy of the GAP6 model. However, gender was not a significant predictor in the KICO registry, and other predictors might be useful. Further research considering predictors other than the GAP variables is needed in the near future. Forth, about half patients in KICO registry were included in this study due to missing data of 6MWT. The missing data was concentrated in the early period of enrollment, and careful interpretation is required to possible selection bias.

## Conclusion

The GAP model is a valuable tool for determining prognosis in patients with IPF. However, the GAP model did not accurately predict the 3-year mortality rate among patients in the KICO registry, and the calibration at 3 years was not satisfactory. Therefore, we designed the GAP6 model by adding nadir SpO2 as a new risk-assessment system. The GAP6 model improves the prediction ability with C-index and AUC; however, additional multinational research is needed to confirm these findings and validate the applicability and accuracy of this risk-assessment system.

## Data availability statement

The original contributions presented in the study are included in the article/Supplementary material, further inquiries can be directed to the corresponding author.

## Ethics statement

The studies involving human participants were reviewed and approved by Institutional Review Board of Haeundae-Paik Hospital. Written informed consent for participation was not required for this study in accordance with the national legislation and the institutional requirements.

## Author contributions

MP had full access to all of the data in the study and takes responsibility for the integrity of the data and the accuracy of the data analysis. SC, YK, YK, SP, JP, YJ, JoL, S-TU, T-HK, YK, and BS contributed substantially to the study design. H-kL, S-HY, HL, S-HK, E-JL, HC, HK, EH, and W-YL dedicated to data analysis and interpretation. JaL, JJ, H-JJ, SK, MC, HY, SJ, JS, and HL contributed to the writing of the manuscript. All authors participated in the interpretation of the data, shared critical feedback and provided final approval for submission.

## Conflict of interest

The authors declare that the research was conducted in the absence of any commercial or financial relationships that could be construed as a potential conflict of interest.

## Publisher’s note

All claims expressed in this article are solely those of the authors and do not necessarily represent those of their affiliated organizations, or those of the publisher, the editors and the reviewers. Any product that may be evaluated in this article, or claim that may be made by its manufacturer, is not guaranteed or endorsed by the publisher.

## Footnotes

^1^http://IPF.crf.kr
