# Supplementary material for: New prognostic scoring system for mortality in idiopathic pulmonary fibrosis by modifying the gender, age, and physiology model with desaturation during the six-minute walk test
Source: Front Med (Lausanne). 2023 Jan 25;10:1052129. doi: 10.3389/fmed.2023.1052129 (PMC9905836; doi:10.3389/fmed.2023.1052129)
Supplement: Supplementary file 1 [file Presentation_1.ZIP › Supplementary materials.docx]

Supplementary Material

## Supplementary Figures

**
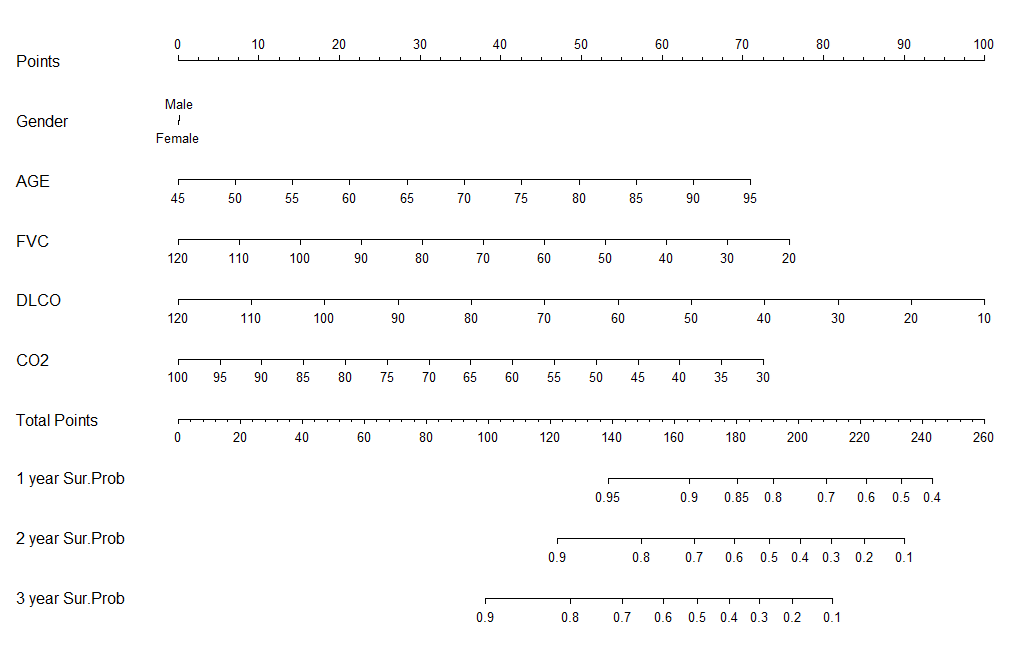
**

**Supplementary Figure 1.** **Nomogram with point-linear predictor unit mapping and total point-survival probability mapping for overall survival in multivariate Cox analysis.**

FVC, forced vital capacity; DLco, diffusing capacity of the lung for carbon monoxide; CO_2_, carbon dioxide; Sur.Prob, survival probability

**
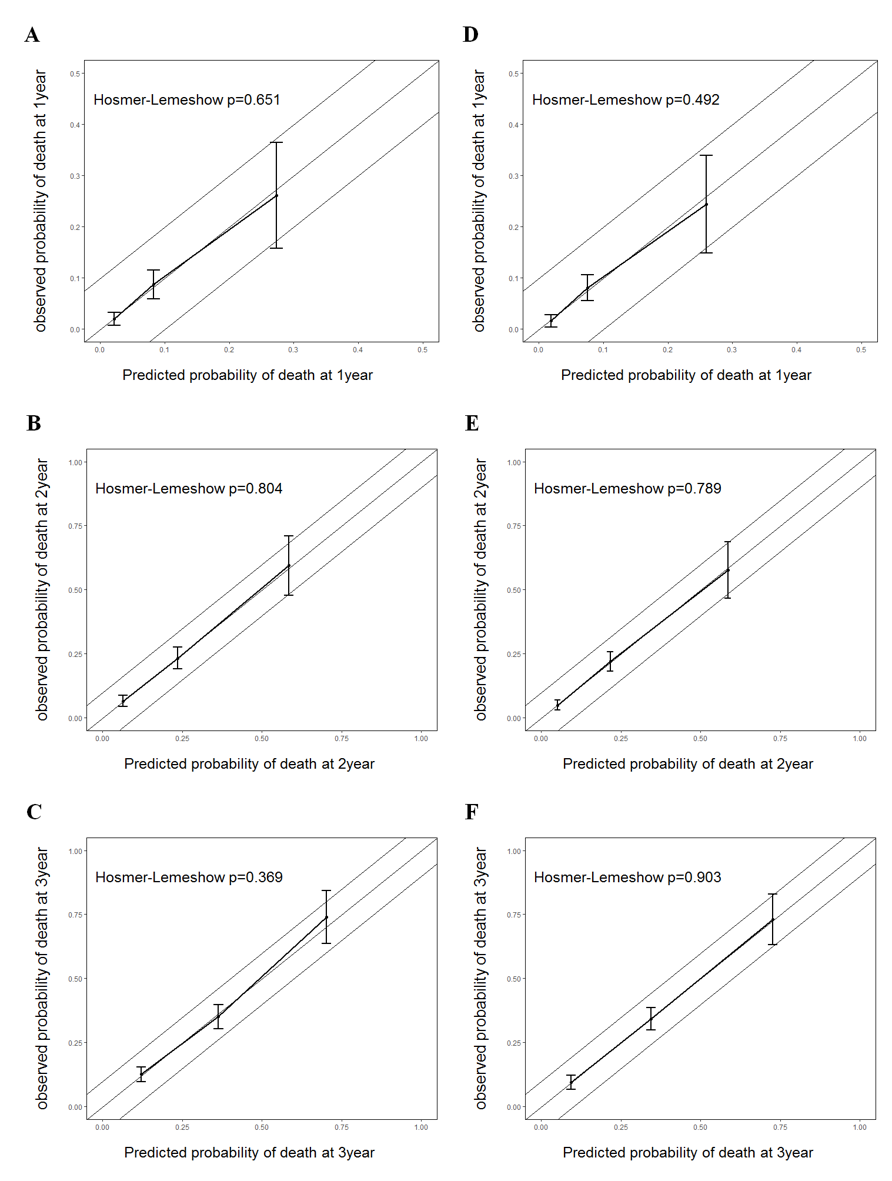
**

**Supplementary Figure 2.** **Comparison of the risk of death predicted by the GAP and GAP6 models with the observed mortality using Hosmer-Lemeshow test.**

(A), (B), and (C): Predicted and observed probability of death at 1, 2, and 3 years by the GAP model, respectively

(D), (E), and (F): Predicted and observed probability of death at 1, 2, and 3 years by the GAP6 model, respectively

GAP, gender, age, and lung physiology; GAP6, gender, age, and lung physiology with desaturation during six-minute walk test
