# Supplementary figures and images for: New prognostic scoring system for mortality in idiopathic pulmonary fibrosis by modifying the gender, age, and physiology model with desaturation during the six-minute walk test
Source: Front Med (Lausanne). 2023 Jan 25;10:1052129. doi: 10.3389/fmed.2023.1052129 (PMC9905836; doi:10.3389/fmed.2023.1052129)

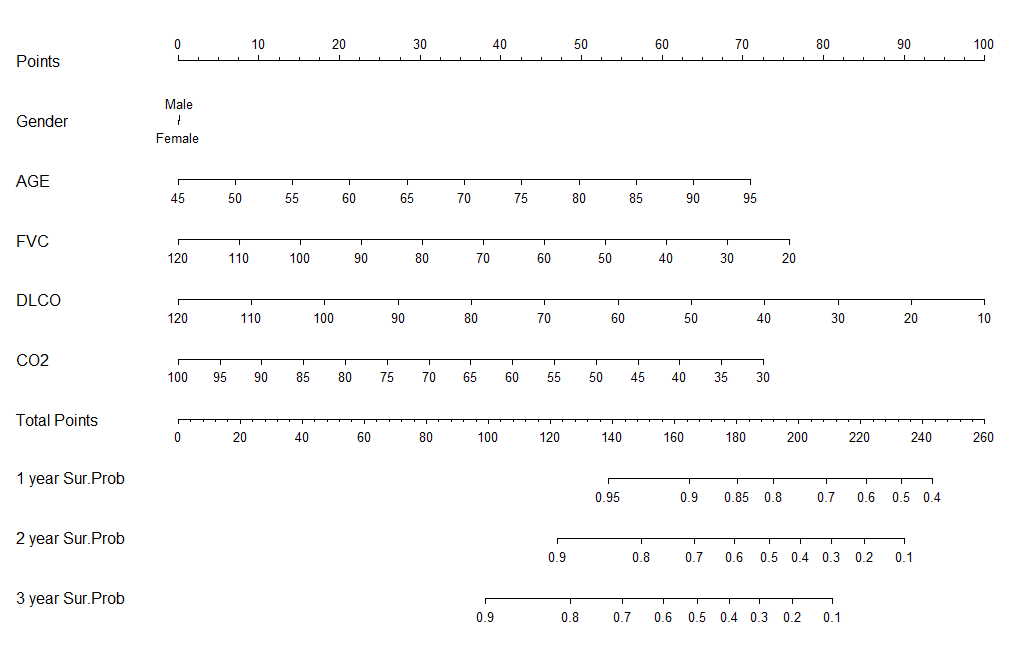

Supplement: Supplementary file 1 [file Presentation_1.ZIP › Supplementary figure 1.tif]

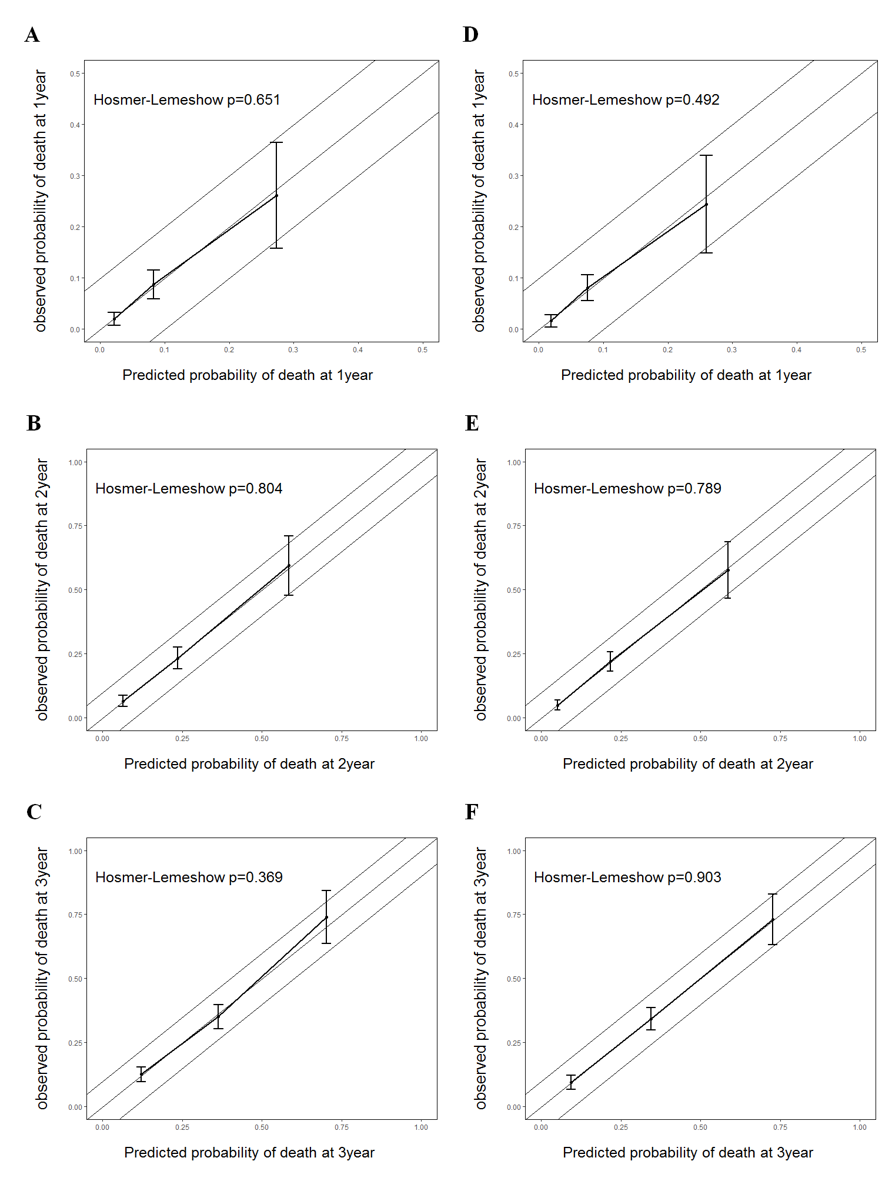

Supplement: Supplementary file 1 [file Presentation_1.ZIP › Supplementary figure 2.tif]
